# Emergency laparotomy in the older patient: factors predictive of 12-month mortality—Salford-POPS-GS. An observational study

**DOI:** 10.1007/s40520-020-01578-0

**Published:** 2020-05-24

**Authors:** Arturo Vilches-Moraga, Mollie Rowley, Jenny Fox, Haroon Khan, Areej Paracha, Angeline Price, Lyndsay Pearce

**Affiliations:** 1 Ageing and Complex Medicine Department, Salford Royal NHS Foundation Trust, Stott Lane, Salford, Manchester, M6HD8 UK; 2 General Surgery Department, Salford Royal NHS Foundation Trust, Stott Lane, Salford, Manchester, M6HD8 UK

**Keywords:** Emergency laparotomy, Frailty, Older people, Mortalitty, Comprehensive geriatric assessment, Surgery

## Abstract

**Introduction:**

Although high rates of in-hospital mortality have been described in older patients undergoing emergency laparotomy (EL), less is known about longer-term outcomes in this population. We describe factors present at the time of hospital admission that influence 12-month survival in older patients.

**Methods:**

Observational study of patients aged 75 years and over, who underwent EL at our hospital between 8th September 2014 and 30th March 2017.

**Results:**

113 patients were included. Average age was 81.9 ± 4.7 years, female predominance (60/113), 3 (2.6%) lived in a care home, 103 (91.2%) and 79 (69.1%) were independent of personal and instrumental activities of daily living (ADLs) and 8 (7.1%) had cognitive impairment. Median length of stay was 16 days ± 29.9 (0–269); in-hospital mortality 22.1% (25/113), post-operative 30-day, 90-day and 12-month mortality rates 19.5% (22), 24.8% (28) and 38.9% (44). 30-day and 12-month readmission rates 5.7% (5/88) and 40.9% (36). 12-month readmission was higher in frail patients, using the Clinical Frailty Scale (CFS) score (64% 5–8 vs 31.7% 1–4, *p* = 0.006). Dependency for personal ADLs (6/10 (60%) dependent vs. 38/103 (36.8%) independent, *p* = 0.119) and cognitive impairment (5/8 (62.5%) impaired vs. 39/105 (37.1%) no impairment, *p* = 0.116) showed a trend towards higher 12-month mortality. On multivariate analysis, 12-month mortality was strongly associated with CFS 5–9 (HR 5.0403 (95% CI 1.719–16.982) and ASA classes III–V (HR 2.704 95% CI 1.032–7.081).

**Conclusion:**

Frailty and high ASA class predict increased mortality at 12 months after emergency laparotomy. We advocate early engagement of multi-professional teams experienced in perioperative care of older patients.

## Introduction

The UK population is advancing in age and, consequently, the age of the average surgical patient is increasing. By 2041, the “baby boom” generation will be well into their 70 s and the population aged 85 years and over is predicted to double [1]. Half of all patients receiving emergency laparotomy are aged 70 years and older [2].

Increasing age is associated with multimorbidity, reduced physiological ability to accommodate the stress of surgery and higher rates of mortality and morbidity [3–6]. Surgical collaboration with medical care of the older person (MCOP) teams is an audit standard for the National Emergency Laparotomy Audit (NELA) 2. To date, this need has remained considerably unmet with input only provided in 19% of cases 2.

Management of the older surgical patient and assessment of perioperative risk warrants holistic comprehensive evaluation of multiple characteristics including disability and pre- and post-operative functional status. These elements are not routinely measured in older patients undergoing emergency laparotomy [7]. The Perioperative care of the Older Patient in Surgery-General Surgery (POPS-GS) teams, such as in our institution, record Rockwood Clinical Frailty Score (CFS) score, and functional, cognitive and demographic data for this vulnerable patient group [8].

The presence of frailty in patients undergoing emergency surgical laparotomy is associated with greater risks of ninety-day mortality and postoperative morbidity [9]. To the best of our knowledge, this study is the first to describe the long-term impact of frailty and impairments in functional status, mobility and cognition in older patients undergoing emergency laparotomy.

## Methods

All patients aged 75 years or older undergoing emergency laparotomy at an urban teaching hospital between 8th September 2014 and 30th March 2017 were included in this prospective study.

Patients were identified post-operatively on assessment by the POPS-GS team. 25/113 patients were not directly reviewed by the POPS-GS team and identified through data collection for the National Emergency Laparotomy Audit, and analysed to allow for more accurate reflection of outcomes. The only exclusion criterion was to remain an inpatient > 90 days prior to the final date of data collection. Patients who had more than one laparotomy on separate admissions were only included for index admission.

Data collected included indication for surgery, American Society of Anaesthetists (ASA) class, in-hospital mortality and length of stay. As part of the POPS-GS team’s assessment, all patients had functional status established and documented on admission including residence, dependency on activities of daily living, mobility, cognition and continence. For patients not reviewed by the POPS-GS team, the electronic patient record was reviewed by an independent researcher to gain this information. Frailty was measured using Rockwood’s Clinical Frailty Scale (CFS) [10]. Each patient was graded from 1 to 9, where 1–4 were not frail and 5–9, frail.

The Katz index [11] was employed to record activities of daily living (ADLs). An individual was defined as dependent on personal ADLs if they required assistance for personal care such as washing and dressing. The Lawton scale [12] defined criteria for dependency on instrumental ADLs with examples of instrumental ADLs including shopping, housekeeping and food preparation. Patients were characterised as having mobility impairment if they mobilised with a walking frame, assistance of others, transferred from bed to chair only, or were completely bed-bound. Electronic patient records were then prospectively reviewed by the POPS team at 6-monthly intervals to monitor mortality and rates of readmission up to 1st April 2018.

Multivariate and univariate analyses were performed using Microsoft Excel and SPSS, Inc., Chicago, IL.

The institutional ethics committee did not require ethical approval as data were collected as part of routine care and deemed service evaluation.

## Results

113 patients were included in this study. Of these, 88 patients received input by the POPS-GS team. Overall, the mean age was 81.9 ± 4.65 years; there was a female predominance at 53.1% and 49 (43.3%) were ASA class I or II. The majority of the population was independent with 103 (91.2%) and 79 (69.1%) patients independent of personal and instrumental ADLs, respectively. Only 2.7% of the cohort was admitted from a care home. 97 (83.2%) mobilised independently with a stick or no aid, and cognitive impairment was present in 8 (7.10%). 104 patients (92%) and 109 patients (96.4%), respectively, were continent of urine and faeces. Increasing age was significantly associated with impaired mobility (*p* < 0.001) and impaired cognition (*p* = 0.001).

Bowel obstruction and perforation were the most common diagnoses resulting in 42 (46%) of laparotomies. Complications of hernias were also common, affecting 22.1% of individuals. Increasing age was significantly associated with higher incidence of complications of hernias (*p* = 0.003). Peritonitis was present in 6 (5.3%) cases and was associated with increased in-hospital mortality, with 5/6 (83.3%) not surviving hospital admission.

Median LOS was 16 ± 29.9 days (0–269) and 5 (4.4%) patients remained in hospital at 60 days following laparotomy. In-hospital mortality rate was 22.1% (25) with 30- and 90-day mortality rates of 19.5% (22) and 24.8% (28), respectively. At 12 months, the mortality rate was 38.9% (44). 30-day and 12-month rehospitalisation rates were 5.6% (5/88) and 40.9% (36/88), with proportionally higher rates of readmission with increasing age. Median time to readmission was 176 days, with proportionally higher rates of readmission in frail patients (*p* = 0.006).

**Fig. 1:**
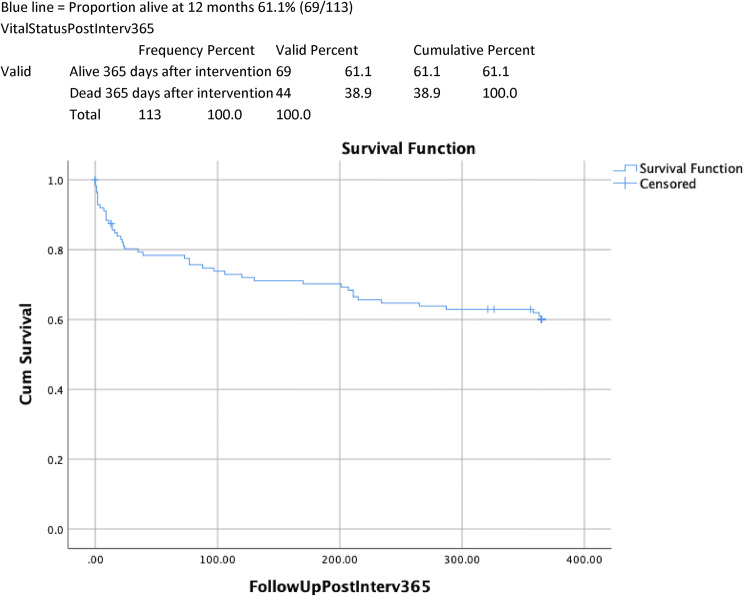
Overall 12-month survival after emergency laparotomy

**Fig. 2:**
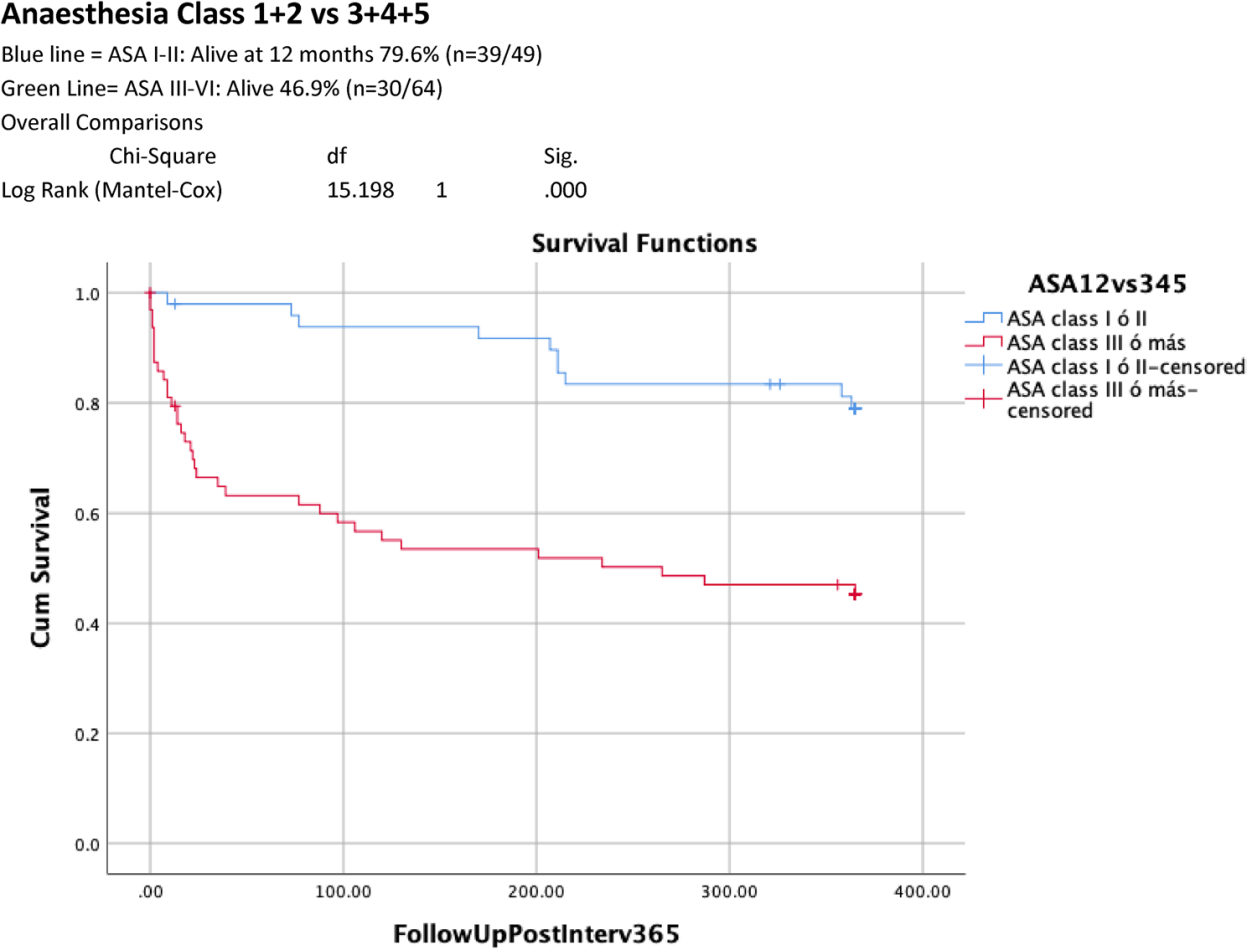
12-month survival after emergency laparotomy according to American Society Anaesthesia Class 1 + 2 vs 3 + 4 + 5

**Fig. 3:**
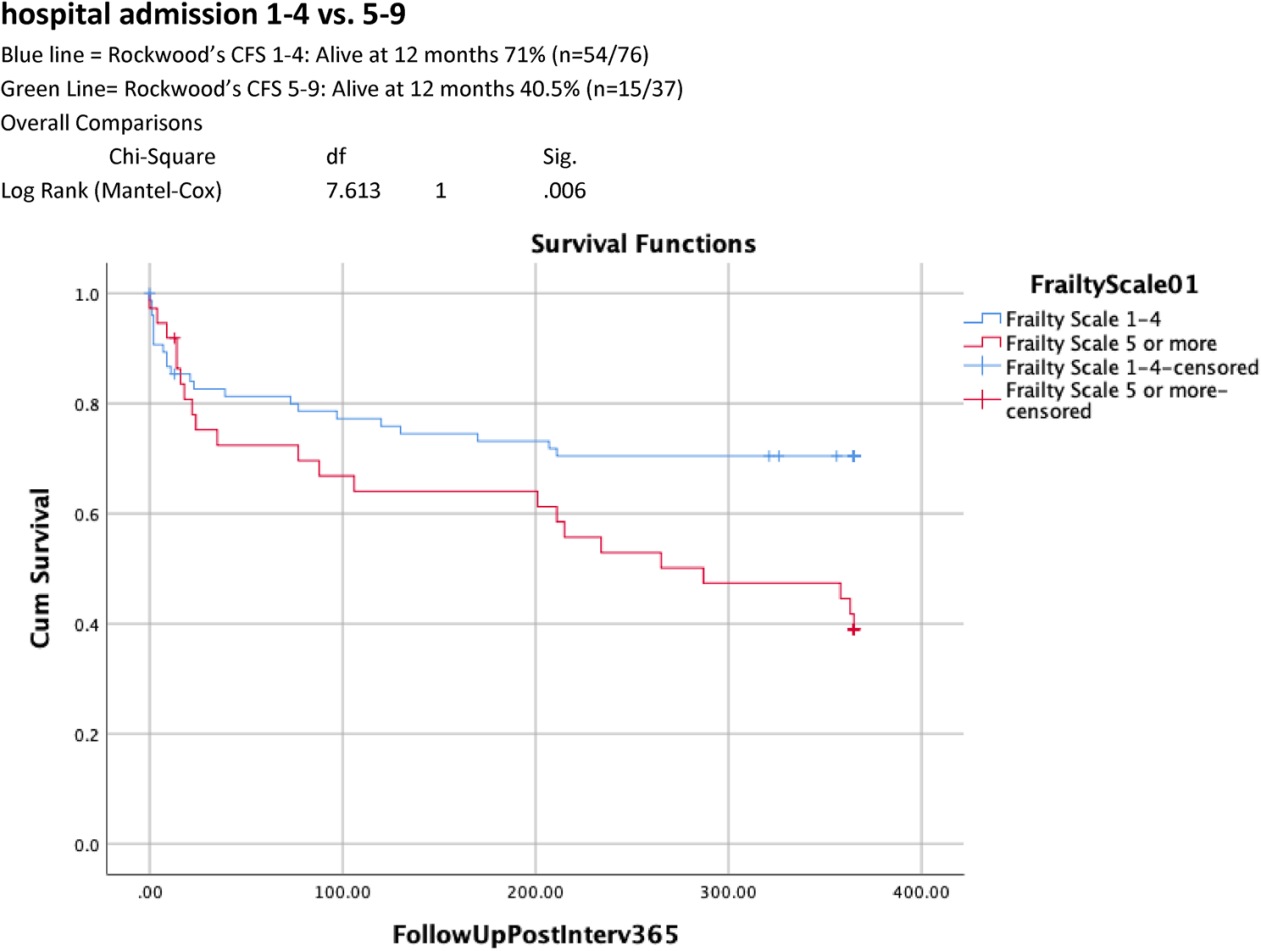
12-month survival after emergency laparotomy according to Clinical Frailty at the time of hospital admission 1–4 vs. 5–9

Table 1 shows univariate analysis of factors predictive of 12-month survival. 12-month mortality rate was significantly higher in those with an ASA classes III–V compared to those with ASA classes I–II (34/64 (53.1% v 10/49(20.4%), *p* < 0.001). Increasing age was associated with higher ASA score with 5/6 of those aged 90 years and above identified as ASA III–V. CFS scores of 5–9 were associated with significantly higher 12-month mortality (59.5% v 28.9%, *p* = 0.002) and readmission rates (64% vs 31.7%, *p* = 0.006) when compared with CFS scores of 1–4. Dependency for personal ADLs (6/10 (60%) dependent v 38/103 (36.8%) independent, *p* = 0.0.119) and cognitive impairment (5/8 (62.5%) impaired vs 39/105 (37.1%) no impairment, *p* = 0.116), were associated with a trend towards higher mortality at 12 months. Kaplan–Meier curves of 12-month cumulative survival post-intervention (Figs. 1, 2, 3) for all patients illustrate 12-month survival in this cohort and also according to ASA class and CFS score.

**Table 1:** Univariate analysis: factors associated with increased mortality 12 months after emergency laparotomy

	Total (*n* = 113)	Alive 61.6% (*n* = 69)	Dead 38.9% (*n *= 44)	*p* value
Age in years (mean ± SD)	81.9 ± 4.65	81.5 ± 4.42	82.3 ± 5.0	0.389
Age (years)				
< 80	44.2% (50)	43.5% (30)	45.5% (20)	0.138
80–89	50.4% (57)	53.6% (37)	45.5% (20)	
≥ 90	5.3% (6)	2.9% (2)	9.1% (4)	
Female	53% (60)	53.6% (37)	52.3% (23)	0.979
Male	47% (53)	46.4% (32)	47.7% (21)	
Dependent personal ADLs	8.80% (10)	5.8% (4)	13.6% (6)	0.119
Independent personal ADLs	91.1% (103)	94.2% (65)	86.4% (38)	
Dependent instrumental ADLs	30.1% (34)	23.2% (16)	40.9% (18)	0.071
Independent instrumental ADLs	69.9% (79)	76.8% (53)	59.1% (26)	
Dependent mobility	16.8% (19)	52.6% (10)	47.3% (9)	0.282
Independent mobility	83.2% (94)	62.7% (59)	37.2% (35)	
Cognitive impairment	7.10% (8)	37.5% (3)	62.5% (5)	0.116
No cognitive impairment	92.9% (105)	62.8% (66)	37.1% (39)	
ASA Classes III–V	56.6% (64)	46.8% (30)	53.1% (34)	< 0.001
ASA Classes I–II	43.4% (49)	79.5% (39)	20.4% (10)	
Clinical Frailty Scale 5–9	32.7% (37)	40.5% (15)	59.9% (22)	0.002
Clinical Frailty Scale 1–4	67.3% (76)	71.1% (54)	28.9% (22)	
Urinary Incontinence	7.9% (9)	66.6% (6)	33.3% (3)	0.088
No Urinary Incontinence	89.3% (101)	60.3% (61)	39.6% (40)	
Nursing/Residential home	2.70% (3)	66.6% (2)	33.3% (1)	0.041
Non-institutionalised	97.3% (110)	60.9% (67)	39% (43)	
Bowel obstruction/perforation	46.01% (52)	59.6% (31)	40.3% (21)	0.771
Liver/Biliary conditions	6.20% (7)	100% (7)	0% (0)	0.028
Hernias	22.1% (25)	64% (16)	36% (9)	0.461
Peritonitis	5.30% (6)	16.6% (1)	83.3% (5)	0.032
Miscellaneous diagnoses	5.30% (6)	66.6% (4)	33.3% (2)	0.567
Gastrointestinal ulcers	0.90% (1)	0% (0)	100% (1)	0.389
Diverticulitis	2.70% (3)	66.6% (2)	33.3% (1)	0.665
Bowel ischemia	3.50% (4)	50% (2)	50% (2)	0.508
Cancer (curative intent)	6.20% (7)	71.4% (5)	28.5% (2)	0.44
Cancer (progression**)**	1.80% (2)	50% (1)	50% (1)	0.629

*ADLs* activities of daily living, *ASA* American Society of Anesthetists Physical Status

Multivariate analysis (Table 2) showed that 12-month mortality was strongly associated with ASA classes III–V (Hazard Ratio 2.704 95% CI 1.032–7.081, *p* = 0.043) and CFS score of 5–9 (Hazard Ratio 5.0403 (95% CI 1.719–16.982, *p* = 0.004).

**Table 2:** Multivariate analyses: factors associated with increased mortality 12 months after emergency laparotomy

	Wald	Sig	Exp(B)	CI. 95% EXP(B)	
ASA Classes 1–2 vs 3–5	4.098	0.043	2.704	1.032	7.081
CFS 1–4 vs 5–9	8.337	0.004	5.403	1.719	16.982
Reduced mobility	2.087	0.149	0.200	0.022	1.776
No POPS-GS	11.234	0.001	6.620	2.192	19.993

*ASA* American Society of Anesthetists Physical Status, *CFS* Clinical Frailty Score, *POPS = GS* Perioperative Care of Older Persons-General Surgery

## Discussion

Our study reports those factors present at the time of hospital admission that are predictive of increased 12-month mortality in older patients following emergency laparotomy. According to multivariate analysis, the presence of clinical frailty, as evidenced by a Clinical Frailty Score of 5 or above, was the strongest predictive factor, surpassing others indicators such as high ASA class. Previous research reported increased mortality in frail patients at 30 and 90 days [13], and retrospectively at 12 months [14], but to date this is the first study communicating long-term prospective frailty data.

We chose Rockwood’s Clinical Frailty Scale from a large range of frailty assessment tools for a variety of reasons. Not only has this score has been validated in UK patients, it has also previously been utilised in acute hospital settings, is easy to use (takes less than 1 min to conduct, no equipment and little training) and easily reproducible. There are other examples of frailty indexes alongside objective measures such as grip strength and gait speed [15]. Clinical significance of such measures in terms of predicting surgical outcome has not yet been satisfactorily been evaluated, and this is an area of continuing research.

Older patients tend towards multimorbidity and polypharmacy, and advanced age influences mortality in both high- and low-risk surgeries. In one 5-year study of 100 emergency laparotomy patients aged 80 years and older, a 45% in-hospital mortality rate was demonstrated with sepsis the leading cause of death [16].

The most common indication for surgery in our cohort was bowel obstruction secondary to cancer or adhesions, with a 19.2% mortality rate. This is consistent with previous literature as the modal indication for emergency laparotomy. A large study of 105,000 surgical admissions aged 70 years or above from the North East of England, with arguably the most similar patient population, found similar rates of mortality for intestinal obstruction at 22%, which was the second highest total mortality rate after intestinal vascular disorder [7]. Other than peritonitis (*p* = 0.032), no other diagnosis was associated with statistically worse long-term survival. Peritonitis is a marker of severe intra-abdominal pathology and also related to very high rates of in-hospital mortality.

The POPS-GS team does not routinely offer in-reach into high-dependency areas as it is felt that at such acute levels of care, geriatrician-led support would in most cases provide little merit. However, a large proportion of patients died in high-dependency care prior to POPS-GS review raising the question as to whether these individuals were ideal candidates for theatre in the first instance and, further to this, if these deaths could have been foreseen and surgery avoided. In the immediate period following step-down to the general surgical ward patients are at increased risk of morbidity and mortality. There is evidence to suggest that this is seen more frequently amongst individuals with significant comorbidities and poor performance status [17–19]. Communication between geriatric, surgical and critical care specialities, particularly at the point of transfer to ward-level care, could, therefore, provide benefit.

At present, there is considerable debate regarding the most appropriate risk scoring measurement tools in emergency laparotomy. One single-centre study of patients over 70 years of age found that low-risk P-POSSUM and ASA scores may be able to predict increased likelihood of survival and described a correlation between preoperative lactate and haemoglobin and days to death [20]. A further systematic review of risk assessment in all emergency laparotomy patients found that none of the tools for risk assessment in older patients could provide adequate discrimination of outcomes although APACHE II was generally the best predictor of mortality [21]. Frailty and other age-related syndromes such as sarcopenia are independent predictors of perioperative mortality [22–25]. We have shown in this study that patients who are frail often have worse long-term outcomes in terms of mortality.

The most recent NELA report reported that only around 30% of hospitals in the UK use a frailty score in perioperative assessment of emergency laparotomy [26]. When considering the impact of age and frailty on clinical decision-making for surgery, there are of course far reaching legal and ethical implications. It is important not to discriminate based on age or dependence without justification of significantly worse outcome. A holistic assessment prior to surgery that includes these elements may prove useful in identifying those individuals likely to succumb to the stresses and complications of emergency laparotomy. Timely identification of high-risk patients may in turn enable a less invasive approach including avoiding surgical interventions and admission to critical care, and facilitating high-quality palliative care and a more dignified death.

Most patients included in this study were relatively independent and mobile with low prevalence of pre-admission cognitive impairment. These components of the geriatric assessment did not have a significant predictive value in terms of long-term survival probably due to insufficient statistical power.

The overall fatality at 90 days and 12 months after emergency laparotomy in this cohort were 24.8% and 39.9%, respectively. This poor long-term survival is worse than that of cancer, hospital admission for decompensated heart failure or hip fracture. More than ever before, there is an urgent need for studies aimed at reporting quality of life following emergency laparotomy in this patient group; and raises questions regarding suitability of patients for such high-risk surgery.

Limitations of this research include that this was a single-centre study in a system where the service was developing (as opposed to well developed) over the three years the study was taking place. Because of this, a significantly higher proportion of patients were seen by the team in the third year of the study than in the first, and this may have implications in terms of replicability. Other limitations lie in the remit of action for the POPS-GS team. Our team reviewed most patients post-operatively, with no structured or routine input into critical care environments, unless specific referral was made. The team also provided no insight into patients not admitted to general surgery, for example those managed conservatively for acute abdomen.

## Conclusions and key messages

- In our cohort of older persons undergoing emergency laparotomy, frailty was common.
- Frailty appeared to significantly reduce long-term survival post-intervention.
- More work is needed on identification and assessment of geriatric syndromes such as frailty by non-geriatricians in the perioperative period.
- This study adds to a growing body of evidence for perioperative comprehensive geriatric assessment, risk stratification, collaborative multidisciplinary working, timely rehabilitation and discharge planning.

We advocate a personalised, multidisciplinary and holistic approach for frail individuals considered for emergency laparotomy, with early involvement by care of older person’s teams.
